# Explainable depression detection with multi-aspect features using a hybrid deep learning model on social media

**DOI:** 10.1007/s11280-021-00992-2

**Published:** 2022-01-28

**Authors:** Hamad Zogan, Imran Razzak, Xianzhi Wang, Shoaib Jameel, Guandong Xu

**Affiliations:** 1 University of Technology Sydney (UTS), Sydney, Australia; 2 Jazan University, Jazan, Saudi Arabia; 3 Deakin University, Geelong, Australia; 4 University of Essex, Colchester, UK

**Keywords:** Depression detection, Social network, Deep learning, Machine learning, Explainability

## Abstract

The ability to explain why the model produced results in such a way is an important problem, especially in the medical domain. Model explainability is important for building trust by providing insight into the model prediction. However, most existing machine learning methods provide no explainability, which is worrying. For instance, in the task of automatic depression prediction, most machine learning models lead to predictions that are obscure to humans. In this work, we propose explainable Multi-Aspect Depression Detection with Hierarchical Attention Network **MDHAN**, for automatic detection of depressed users on social media and explain the model prediction. We have considered user posts augmented with additional features from Twitter. Specifically, we encode user posts using two levels of attention mechanisms applied at the tweet-level and word-level, calculate each tweet and words’ importance, and capture semantic sequence features from the user timelines (posts). Our hierarchical attention model is developed in such a way that it can capture patterns that leads to explainable results. Our experiments show that **MDHAN** outperforms several popular and robust baseline methods, demonstrating the effectiveness of combining deep learning with multi-aspect features. We also show that our model helps improve predictive performance when detecting depression in users who are posting messages publicly on social media. **MDHAN** achieves excellent performance and ensures adequate evidence to explain the prediction.

## Introduction

Mental illness is a serious issue faced by a large population around the world. In the United States (US) alone, every year, a significant percentage of the adult population is affected by different mental disorders, which include depression mental illness (6.7%), anorexia and bulimia nervosa (1.6%), and bipolar mental illness (2.6%) [29]. Sometimes mental illness has been attributed to the mass shooting in the US [30], which has taken numerous innocent lives. One of the common mental health problems is depression that is more dominant than other mental illness conditions worldwide [65]. Diagnosis of depression is usually a difficult task because depression detection needs a thorough and detailed psychological testing by experienced psychiatrists at an early stage [42] and it requires interviews, questionnaires, self-reports or testimony from friends and relatives. Moreover, it is very common among people who suffer from depression that they do not visit clinics to ask help from doctors in the early stages of the problem [69].

Individuals and health organizations have shifted away from their traditional interactions, and now meeting online by building online communities for sharing information, seeking and giving the advice to help scale their approach to some extent so that they could cover more affected populations in less time. Besides sharing their mood and actions, recent studies indicate that many people on social media tend to share or give advice on health-related information [18, 32, 39, 43]. These sources provide the potential pathway to discover the mental health knowledge for tasks such as diagnosis, medications and claims. It is common for people who suffer from mental health problems too often “implicitly” (and sometimes even “explicitly”) to disclose their feelings and their daily struggles with mental health issues on social media as a way of relief [3, 36]. Therefore, social media is an excellent resource to automatically discover people who are depressed. While it would take a considerable amount of time to manually sift through individual social media posts and profiles to locate people going through depression, automatic scalable computational methods could provide timely and mass detection of depressed people which could help prevent many major fatalities in the future and help people who genuinely need it at the right moment. Usually, depressed users act differently when they are on social media, producing rich behavioural data, which is often used to extract various features. However, not all of them are related to depression.

**Fig. 1:**
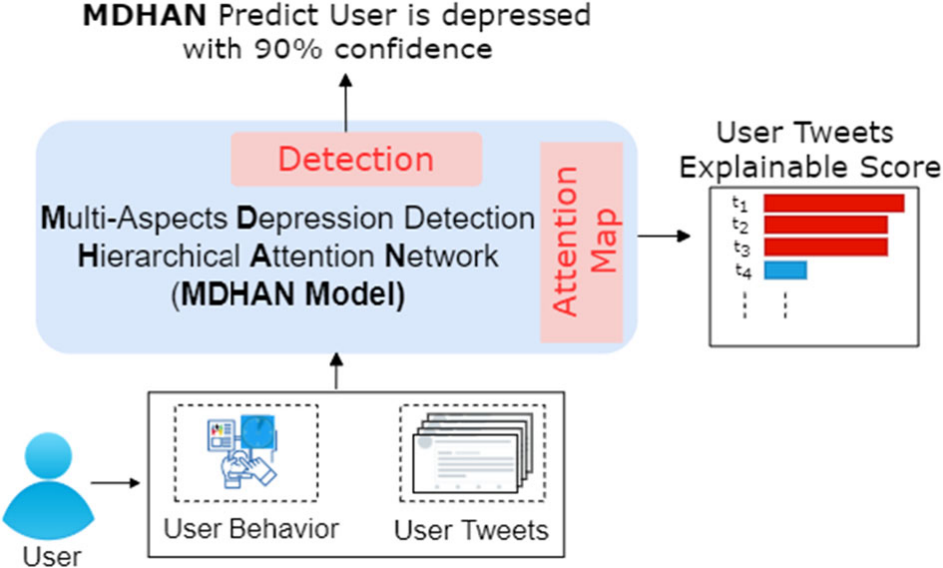
Explainable depression detection

Recently, deep learning has been successfully applied to several application problems, such as stock market predictions [34, 55], traffic flow and traffic accident risk predictions [15, 49, 59], and mental illness detections [23]. Moreover deep learning has been applied for depression detection on social media and showed significantly better performance than traditional machine learning methods. Hamad et al. [68] presented a computational framework for automatic detection of the depressed user that initially selects relevant content through a hybrid extractive and abstractive summarization strategy on the sequence of all user tweets leading to a more fine-grained and relevant content, which then is forwarded to deep learning framework comprising of unified learning machinery of the convolutional neural network coupled with attention-enhanced gated recurrent units leading to better empirical performance than existing strong baseline methods. Even though recent work showed the effectiveness of deep learning methods for depression detection, most of the existing machine learning methods provide no explainability for depression prediction, hence their predictions are obscure to humans which reduces the trust in the deep learning models. An explainable model provides insights into how a deep learning model can be improved and supports understanding. Thus, to engenders the appropriate user trust and provide the reason behind the decision, we aim to develop an explainable deep learning-based solution for depression detection by utilizing multi-aspect features from the diverse behaviour of the depressed user in social media. Apart from the latent features derived from lexical attributes, we notice that the dynamics of tweets, i.e. tweet timeline provides a crucial hint reflecting depressed user emotion change over time. To this end, we propose a hybrid model, **M** ulti-aspect **D** epression Detection **H** ierarchical **A** ttention **N** etwork **MDHAN** to boost the classification of depressed users using multi-aspect features and word embedding features. Figure 1 illustrate the effectiveness of explainability in improving user trust. The model can derive new deterministic feature representations from training data and produce superior results for detecting depression-level of Twitter users, and derive explanations from a user posts content. Besides, we also studied the performance of our model when we used the two components of user posts and his multi-aspect features separately. We found that model performance deteriorated when we used only multi-aspect features. We further show when we combined the two attributes, our model led to better performance. Our model is based on explainable depression detection, which can learn explainable information from a user’s tweets. The attention map in Figure 1 returns a user’s tweets with explainable scores where the higher the score, the more likely tweet that is important and contributed to depression classification. To summarize, our study makes the following **key contributions**:

1. a novel explainable depression detection framework using deep learning of the textual, behavioural, temporal, and semantic aspect features from social media. To the best of our knowledge, this is the first work of using multi-aspect of topical, temporal and semantic features jointly with word embeddings in deep learning for depression detection.
2. introducing the prospective of viewing explainability of model for depression detection and building a pipeline aided with explainability based on hierarchical attention networks to explain the prediction of depression detection.
3. Extensive experiments are conducted on benchmark depression twitter dataset, which shows the superiority of our proposed method when compared to baseline methods.

The rest of our paper is organized as follows. Section 2 reviews the related work to our paper, and in Section 3 we formulate our problem, and present our explainable model for detection depression, and describes the different attributes that we extracted for our model. Section 4 reports experiments and results. Finally, Section 5 concludes this paper.

## Related work

In this section, we will discuss closely related literature and mention how they are different from our proposed method. In general, just like our work, most existing studies focus on user behaviour to detect whether a user suffers from depression or any mental illness. We will also discuss other relevant literature covering word embeddings and hybrid deep learning methods which have been proposed for detecting mental health from online social networks and other resources including public discussion forums.

Understanding depression on online social networks could be carried out using two complementary approaches which are widely discussed in the literature, and they are:

- Post-level behavioural analysis
- User-level behavioural analysis

### Post-level behavioural analysis

Methods that use this kind of analysis mainly target the textual features of the user post that is extracted in the form of statistical knowledge such as those based on count-based methods [25]. These features describe the linguistic content of the post which are discussed in [9, 20]. For instance, in [9] the authors propose a classifier to understand the risk of depression. Concretely, the goal of the paper is to estimate that there is a risk of user depression from their social media posts. To this end, the authors collect data from social media for a year preceding the onset of depression from user profiles and distil behavioural attributes to be measured relating to social engagement, emotion, language and linguistic styles, ego network, and mentions of antidepressant medications. The authors collect their data using crowd-sourcing tasks, which is not a scalable strategy, on Amazon Mechanical Turk. In their study, the crowd workers were asked to undertake a standardized clinical depression survey, followed by various questions on their depression history and demographics. While the authors have conducted thorough quantitative and qualitative studies, they are disadvantageous in that it does not scale to a large set of users and does not consider the notion of text-level semantics such as latent topics and semantic analysis using word embeddings. Our work is both scalable and considers various features which are jointly trained using a novel hybrid deep learning model using a multi-aspect features learning approach. It harnesses high-performance Graphics Processing Units (GPUs) and as a result, has the potential to scale to large sets of instances. In Hu et al., [20] the authors also consider various linguistic and behavioural features on data obtained from social media. Their underlying model relies on both classification and regression techniques for predicting depression while our method performs classification, but on a large scale using a varied set of crucial features relevant to this task.

To analyze whether the post contains positive or negative words and/or emotions, or the degree of adverbs [51] used cues from the text, for example, *I feel a little depressed* and *I feel so depressed*, where they capture the usage of the word *“depressed”* in the sentences that express two different feelings. The authors also analyzed the posts’ interaction (i.e., on Twitter (retweet, liked, commented)). Some researchers studied post-level behaviours to predict mental problems by analysing tweets on Twitter to find out the depression-related language. In [41], the authors have developed a model to uncover meaningful and useful latent structure in a tweet. Similarly, in [44], the authors monitored different symptoms of depression that are mentioned in a user’s tweet. In [45], they study users’ behaviour on both Twitter and Weibo. To analyze users’ posts, they have used linguistic features. They used a Chinese language psychological analysis system called TextMind in sentiment analysis. One of the interesting post-level behavioural studies was done by [44] on Twitter by finding depression relevant words, antidepressants, and depression symptoms. In [40] the authors used post-level behaviour for detecting anorexia; they analyze domain-related vocabulary such as anorexia, eating disorder, food, meals and exercises.

### User-level behaviours

There are various features to model users in social media as it reflects overall behaviour over several posts. Different from post-level features extracted from a single post, user-level features extract from several tweets during different times [51]. It also extracts the user’s social engagement presented on Twitter from many tweets, retweets and/or user interactions with others. Generally, posts’ linguistic style could be considered to extract features [20, 64]. The authors in [44] extracted six depression-oriented feature groups for a comprehensive description of each user from the collected data set. The authors used the number of tweets and social interactions as social network features. For user profile features, they have used user shared personal information in a social network. Analysing user behaviour looks useful for detecting eating disorders. In Wang et al., [57] they extracted user engagement and activities features on social media. They have extracted linguistic features of the users for psychometric properties which resembles the settings described in [40, 45] where the authors have extracted 70 features from two different social networks (Twitter and Weibo). They extracted features from a user profile, posting time and user interaction features such as several followers and followee. Similarly, Wong et al. combined user-level and post-level semantics and cast their problem as multiple instances learning setups. The advantage that this method has is that it can learn from user-level labels to identify post-level labels [61].

Recently, Lin et al. [12] applied a CNN-based deep learning model to classify Twitter users based on depression using multi-modal features. The framework proposed by the authors has two parts. In the first part, the authors train their model in an offline mode where they exploit features from Bidirectional Encoder Representations from Transformers (BERT) and visual features from images using a CNN model. The two features are then combined, just as in our model, for joint feature learning. There is then an online depression detection phase that considers user tweets and images jointly where there is a feature fusion at a later stage. In another recently proposed work [6], the authors use visual and textual features to detect depressed users on Instagram posts than Twitter. Their model also uses multi-modalities in data, but keep themselves confined to Instagram only. While the model in [26] showed promising results, it still has a certain disadvantage. For instance, BERT vectors for masked tokens are computationally demanding to obtain even during the fine-tuning stage, unlike our model which does not have to train the word embeddings from scratch. Another limitation of their work is that they obtain sentence representations from BERT, for instance, BERT imposes a 512 token length limit where longer sequences are simply truncated resulting in some information loss, where our model has a much longer sequence length which we can tune easily because our model is computationally cheaper to train. We have proposed a hybrid model that considers a variety of features, unlike these works. While we have not specifically used visual features in our work, using a diverse set of crucial relevant textual features is indeed reasonable than just visual features. Of course, our model has the flexibility to incorporate a variety of other features including visual features.

Multi-modal features from the text, audio, images have also been used in [67], where a new graph attention-based model embedded with multi-modal knowledge for depression detection. While they have used the temporal CNN model, their overall architecture has experimented on small-scale questionnaire data. For instance, their dataset contains 189 sessions of interactions ranging between 7–33 min (with an average of 16 min). While they have not experimented with their method with short and noisy data from social media, it remains to be seen how their method scales to such large collections. Xezonaki et al., [62] propose an attention-based model for detecting depression from transcribed clinical interviews than from online social networks. Their main conclusion was that individuals diagnosed with depression use affective language to a greater extent than those who are not going through depression. In another recent work [60], the authors discuss depression among users during the COVID-19 pandemic using LSTM and fastText [31] embeddings. In [46], the authors also propose a multi-model RNN-based model for depression prediction but apply their model on online user forum datasets. Trotzek et al., [50] study the problem of early detection of depression from social media using deep learning where they leverage different word embeddings in an ensemble-based learning setup. The authors even train a new word embedding on their dataset to obtain task-specific embeddings. While the authors have used the CNN model to learn high-quality features, their method does not consider temporal dynamics coupled with latent topics, which we show to play a crucial role in overall quantitative performance. Farruque et al., [16] study the problem of creating word embeddings in cases where the data is scarce, for instance, depressive language detection from user tweets. The underlying motivation of their work is to simulate a retrofitting-based word embedding approach [17] where they begin with a pre-trained model and fine-tune the model on domain-specific data.

Opinions and emotions play an important role in detecting depression in social media product feedback, services, and other topics. The analysis of emotions in users’ posts has continued to be one of the leading research directions. Prior researches [9, 47, 66] have investigated how emotions and affective states play a role in people’s interactions with technology. Recent research in depression identification has shown that excessive self-focused language and negative emotions are key indicators for detecting depressed people [1, 52]. De Choudhury et al. [8] collected a Twitter dataset that included postings from people who had been diagnosed with depression. They studied the sentiment, emotion and linguistic of these tweets. They found interesting differences in the usage of words associated with negative emotions for the depressed user’s tweets. Additionally, Twitter data analysis reveals that moms’ emotional expression, social engagement and linguistic style of moms who experience postpartum depression alter before their baby is even born [9].

Recent studies have started to target depressed users online, extracting features representing user behaviours and classifying these features into different groups, such as the number of posts, posting time distribution, and several followers and followee. Peng et al. extracted different features and classified them into three groups, user profile, user behaviour and user text and used multi-kernel SVM for classification [37]. The above-mentioned works have some limitations. They mainly focused on studying user behaviour than taking cues from user-generated content such as the text they share which make it extremely difficult to achieve high performance in classification. These models also cannot work well to detect depressed users at the user level, and as a result, they are prone to incorrect prediction. Our novel approach combines user behaviour with user history posts. Besides, our strategy to select salient content using automatic summarization helps our model only focus on the most important information. Although recent deep learning methods showed significant performance for depression detection, most of the existing models do not explain prediction since explainability and effectiveness could sometimes conflict. The explainable model can provide deep insight into how a deep learning model can be improved and supports understanding. Therefore, to provide some details and explain user tweets or reasons to make a decision functioning clear or easy to understand, we aim to develop an explainable deep learning-based approach for depression detection. Our proposed model utilized multi-aspect features from the diverse behaviour of the depressed user and his posts on social media.

### Explainable deep learning

Deep neural networks help people make better decisions in various industries by producing more accurate and insightful predictions based on vast amounts of data. However, unlike interpretable machine learning methods [13, 56], deep learning models (DNNs) learned representations are typically not interpretable by humans [14]. As a result, understanding the representations acquired by neurones in intermediate levels of DNNs is important to the explanation of deep neural networks (DNNs) [27, 28]. Meanwhile, concerns about the nature and operation of the deep neural network’s black box have grown, driving an increase in curiosity in deconstructing its essential components and understanding its functions. Therefore, explainability has lately received a lot of attention, owing to the requirement to explain the internal mechanics of a deep learning system [5, 63]. Many recent studies have focused on improving the transparency of deep neural networks to be adequately understood and be reliable. Attention-based methods can improve model transparency and have shown to be effective in various Natural Language Processing (NLP) tasks, including entity recognition, machine translation systems and text classification [2, 58]. Moreover, for document classification [63] and time series forecasting and classification [54], a variety of approaches for designing explainable neural networks employing attention processes have been investigated. In this paper, we propose using hierarchical attention to improve depression detection by capturing the explainability of depressed user tweets.

## Explainable deep depression detection

Suppose we have a set *U* of labelled users from both depression or non-depression samples. Let *A* be a user posts *A* = [*t*_1_,*t*_2_,....,*t*_*L*_] consisting *L* tweets, where *L* is the total number of tweets per user, each tweet *t*_*i*_ contains n-words *t*_*i*_ = [*w*_*i*1_,*w*_*i*2_,....,*w*_*iN*_] where *N* is the total number of words per tweet. Let *M* be the features in total for a user {*m*_*i*_}$^{M}_{i=1}$, and let {1,2,...,*S*} be a finite set of available aspects features, so we denote *M*_*s*_ as the dimension of *S*^*th*^ aspect. Therefore, once we have a user tweets *A* and a set of related user behaviours feature *M*. Our depression detection function is represented as follows:

1 $$ f (A,M) \rightarrow \hat{y} $$

**Fig. 2:**
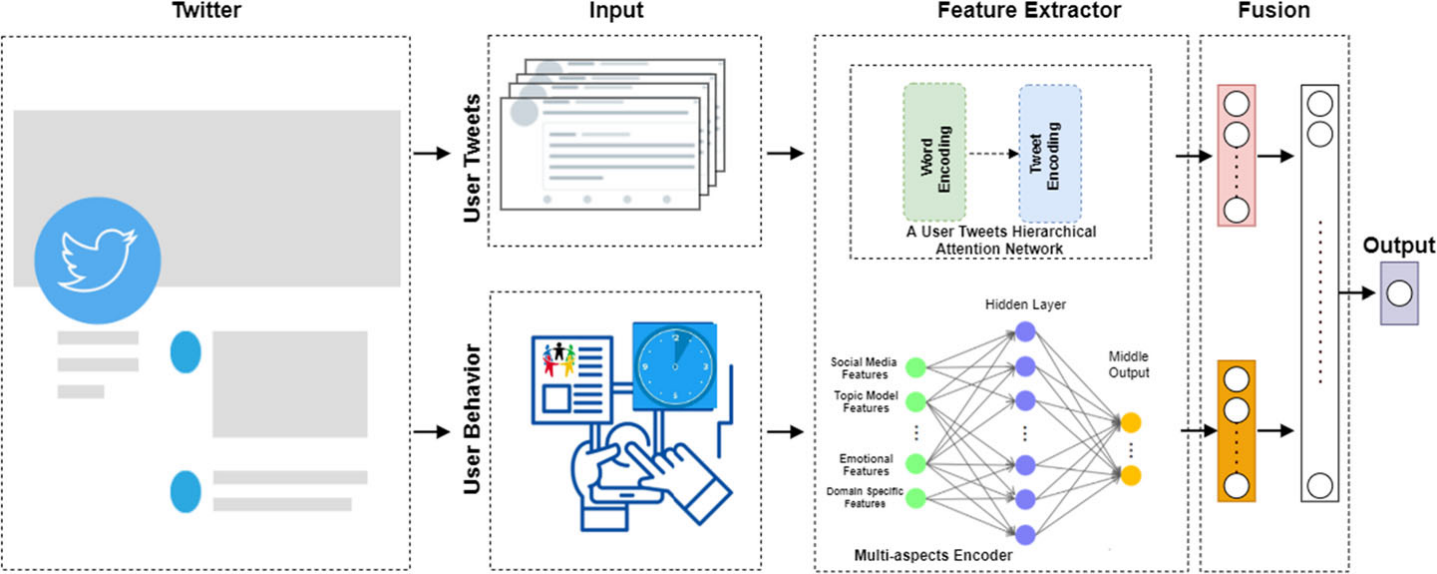
Overview of our proposed model MDHAN: We predict depressed user by fusing two kinds of information: (1) User tweets. (2) User Behaviours

The model has been designed in such a way that it maximizes prediction accuracy. In our problem, we treat depression detection as the binary classification problem, i.e., user can be depressed ($\hat {y} = 1$) or not-depressed ($\hat {y} = 0$). Due to the complexity of user posts and the diversity of their behaviour on social media, we propose a hybrid model based on Hierarchical Attention Networks (HAN) that combines with Multilayer Perceptron (MLP) to detect depression through social media as depicted in Figure 2. For each user, the model takes two inputs for the two attributes. First, the four aspects feature input that represents the user behaviour vector runs into MLP, capturing distinct and latent features and correlation across the features matrix. The second input represents each user input tweet that will be replaced with its embedding and fed to Hierarchical Attention Networks (HAN) to learn some representation features through a hierarchical word and tweet level encoding. The output in the middle of both attributes is concatenated to represent one single vector feature that fed into an activation layer of sigmoid for prediction. In the following sections, we will discuss the following two existing separate architectures.

### Feature selection

From the depression criteria and online behaviours on social media, we extracted a comprehensive set of depression-oriented features inspired by offline symptoms. Each feature group represents a single aspect. While we did not exploit multimedia features such as images or videos, we used a rich set of features to model multiple aspect. We introduce this attribute type where the goal is to calculate the attribute value corresponding to each features aspect for each user. We mainly consider four major aspects as listed below. These features are extracted respectively for each user as follows:

#### Social information and interaction

From this attribute, we extracted several features embedded in each user profile. These are features related to each user account as specified by each feature name. Most of the features are directly available in the user data, such as the number of users following and friends, favourites, etc.

Moreover, the extracted features relate to user behaviour on their profile. For each user, we calculate their total number of tweets, the total length of all tweets and the number of retweets. We further calculate posting time distribution for each user, by counting how many tweets the user published during each of the 24 hours a day. Hence it is a 24-dimensional integer array. To get posting time distribution for each tweet, we extract two digits as hour information, then go through all tweets of each user and track the count of tweets posted in each hour of the day.

#### Emojis sentiment

Emojis allow users to express their emotions through simple icons and non-verbal elements. It is useful to get the attention of the reader. Emojis could give us a glance at the sentiment of any text or tweet, and it is essential to differentiate between positive and negative sentiment text [35]. User tweets contain a large number of emojis which can be classified into positive, negative and neutral. For each positive, neutral, and negative type, we count their frequency in each tweet. Then we sum up the numbers from each user’s tweets to get the sum for each user. So the final output is three values corresponding to positive, neutral and negative emojis by the user. We also consider Voice Activity Detection (VAD) features. These features contain Valance, Arousal and Dominance scores. For that, we count First Person Singular and First Person Plural. Using affective norms for English words, a VAD score for 1030 words are obtained. We create a dictionary with each word as a key and a tuple of its (valance, arousal, dominance) score as value. Next, we parse each tweet and calculate the VAD score for each tweet using this dictionary. Finally, for each user, we add up the VAD scores of tweets by that user, to calculate the VAD score for each user.

#### Topic distribution

Topic modelling belongs to the class statistical modelling frameworks which help in the discovery of abstract topics in a collection of text documents. It gives us a way of organizing, understanding and summarizing collections of textual information. It helps find hidden topical patterns throughout the process, where the number of topics is specific by the user apriori. It can be defined as a method of finding a group of words (i.e. topics) from a collection of documents that best represent the latent topical information in the collection. In our work, we applied the unsupervised Latent Dirichlet Allocation (LDA) [4] to extract the most latent topic distribution from user tweets. To calculate topic level features, we first consider the corpus of all tweets of all depressed users. Next, we split each tweet into a list of words and assemble all words in decreasing order of their frequency of occurrence, and common English words (stopwords) are removed from the list. Finally, we apply LDA to extract the latent *K* = 25 topics distribution, where *K* is the number of topics. We have found experimentally *K* = 25 to be a suitable value. While there are tuning strategies and strategies based on Bayesian non-parametric [48], we have opted to use a simple, popular, and computationally efficient approach that helps give us the desired results.

#### Domain-specific features

1- Depression symptom counts: It is the count of depression symptoms occurring in tweets, as specified in nine groups in DSM-IV criteria for a depression diagnosis. The symptoms are listed in Appendix A. We count how many times the nine depression symptoms are mentioned by the user in their tweets. The symptoms are specified as a list of nine categories, each containing various synonyms for the particular symptom. We created a set of seed keywords for all these nine categories, and with the help of the pre-trained word embedding, we extracted the similarities of these symptoms to extend the list of keywords for each depression symptom. Furthermore, we scan through all tweets, counting how many times a particular symptom is mentioned in each tweet. 2- Antidepressants: We also focused on the antidepressants, and we created a lexicon of antidepressants from the “Antidepressant” Wikipedia page which contains an exhaustive list of items and is updated regularly, in which we counted the number of names listed for antidepressants. The medicine names are listed in Appendix B.

### User tweets encoder using RNN

**Fig. 3:**
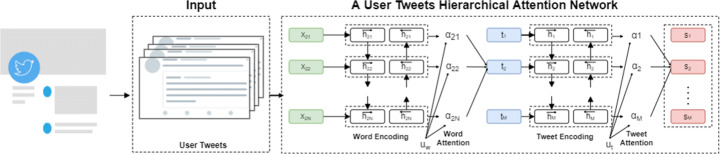
An illustration of hierarchical attention network that we used to encode user tweets

Recently, researchers find that the HAN [10, 63] can generate explanations by considering the most important words and sentences in a document. A depressed user could often have different linguistic style posts, including depressive language use, and mentions of antidepressants and symptoms, which can help detect depression. Additionally, a social media post contains linguistic prompts with different levels of word-level and tweet-level. Every word in a tweet and every tweet of a user is equally important to understand a depressed user in social media. For example, “My dad doesn’t even seem to believe I’m really hurt!”, the word “hurt” contributes more signals to decide whether the tweet is depressed rather than other words in the tweet. So in this way, HAN performs better in predicting the class of given user tweets. Inspired by [63], we proposed Hierarchical Attention Network to learn user tweets representation as depicted in Figure 3. We consider *U* be a user made *M* tweets *T* = [*t*_1_,*t*_2_,....,*T*_*M*_] each tweet *t*_*i*_ = [*w*_1_,*w*_2_,....,*w*_*N*_] contains *N*_*i*_ words. Each tweet is represented by the sequence of d-dimensional embeddings of their words, *t*_*i*_ = [*w*_11_,....,*w*_*MN*_]. And we represent each word as the input layer a fixed-size vector from pre-trained word embeddings.

#### Word encoder

A bidirectional Gated Recurrent Unit (biGRU) is first used as the word level encoder to capture annotations’ contextual information. GRU is a Recurrent Nural Network (RNN) that can capture sequential information and sentences’ long-term dependency. Only two gate functions are used which are reset and update gates. Update gate has been used to monitor the degree to which the previous moment’s status information has been transported into the current state. The higher the update gate value, the more the previous moment’s status information is carried forward. The reset gate has been used to monitor the degree to which the previous moment’s status information is overlooked. The smaller the reset gate value, the more neglected the context will be. Both the preceding and the following words influence the current word in the sequential textual data, so we use the BiGRU model to extract the contextual features. The BiGRU consists of a forward $\overrightarrow {GRU}$ and a backward $\overleftarrow {GRU}$ that are used, respectively, to process forward and backward data. The annotation *w*_*ij*_ represent the word j in a sentence i that contains N-words. Each word of user post (tweet) will convert to a word embedding *x*_*ij*_ utilising GloVe [38].

2 $$ {\overrightarrow{{h}_{ij}^{w}}} = \overrightarrow{GRU} (x_{ij}, \overrightarrow{h_{i(j-1)}}), j \in \{1,..., N\} $$

3 $$ {\overleftarrow{{h}_{ij}^{w}}} = \overleftarrow{GRU} (x_{ij}, \overleftarrow{h_{i(j-1)}}), j \in \{N,..., 1\} $$

The combination of the hidden state that is obtained from the forward GRU and the backward GRU $\overrightarrow {{h}_{ij}^{w}}$ and $\overleftarrow {{h}_{ij}^{w}}$ is represented as ${h}_{ij}^{w} = \left [{\overrightarrow {{h}_{ij}^{w}}} \oplus {\overleftarrow {{h}_{ij}^{w}}}\right ]$. Which carries the complete tweet information centred around *x*_*ij*_.

We describe the attention mechanism. It is crucial to introduce a vector *u*_*ij*_ for all words, which is trainable and expected to capture global words. The ${{h}_{ij}^{w}}$ annotations create the basis for attention that starts with another hidden layer by letting the model learn and randomly initialized biases (*b*_*w*_) and weights (*W*_*w*_) through training. the annotations *u*_*ij*_ will be represented as follows:

4 $$ u_{ij} = \tanh (W_{w} {h}_{ij}^{w} + b_{w}) $$

The product *u*_*ij*_*u*_*w*_ (*u*_*w*_ is randomly initialized) expected to signal the importance of the *j* word and normalized to an importance weight per word *α*_*ij*_ by a softmax function:

5 $$ \alpha_{ij}= \frac{\exp(u_{ij} u_{w})}{\sum\limits_{j}\exp({ij} u_{w})} $$

Finally, a weighted sum of word representations concatenated with the annotations previously determined called the tweet vector *v*_*i*_, where *α*_*t*_ indicating importance weight per word:

6 $$ v_{i}= {\sum}_{t} \alpha_{ij} h_{ij}^{w} $$

#### Tweet encoder

In order to learn the tweet representations ${{h}_{i}^{t}}$ from a learned tweet vector *v*_*i*_, we capture the information of context at the tweet level. Similar to the word encoder component, the tweet encoder employs the same BiGRU architecture. Hence the combination of the hidden state that is obtained from the forward GRU and the backward GRU $\overrightarrow {{{h}_{i}^{t}}}$ and $\overleftarrow {{{h}_{i}^{t}}}$ is represented as ${{h}_{i}^{t}} = \left [{\overrightarrow {{{h}_{i}^{t}}}} \oplus {\overleftarrow {{{h}_{i}^{t}}}}\right ]$. Which capture the coherence of a tweet concerning its neighbouring tweets in both directions. Following that, we want to find user tweets that might explain why someone is sad. They should also help identify depression since they give good explainability. Since a user tweets may not be equally important in determining and explaining whether a user is depressed, we use attention over user tweets to capture the semantic affinity of tweets and learn their attention weights based on their relevance to the depression, allowing more reliable and explainable predictions. we will capture the related tweets in the formed vector $\hat {t}$ by using tweet level attention layer. The product *u*_*i*_*u*_*s*_ is expected to signal the importance of the *i* tweet and normalized to an importance weight per tweet *α*_*i*_. Finally, *s*_*i*_ will be a vector that summarizes all the tweet information in a user post:

7 $$ s_{i}= {\sum}_{t} \alpha_{i} {{h}_{i}^{t}} $$

### Multi-aspect encoder

Suppose the input which resembles a user behaviour be represented as [*m*_1_,*m*_2_,...,*m*_*M*_] where M is the total number of features and *M*_*s*_ is the dimension of *S*^*th*^ aspect. Hence, to obtain fine-grained information from user behaviours features, the multi-aspect features are fed through a one-layer MLP to get a hidden representation *m*_*i*_:

8 $$ p_{i}=f \left( b + \sum\limits_{i=1}^{M} W_{i} m_{i} \right) $$

where *f* stands for the nonlinear function and the outcome of behaviour modelling *p*_*i*_ is the high-level representation that captures the behavioural semantic information and plays a critical role in depression diagnosis.

### Classification layer

At the classification layer, we need to predict whether the user is depressed or not depressed. So far, we have introduced, how we encode user multi aspect behaviours features (*p*) and how we can encode user tweets by modelling the hierarchical structure from word level and tweet level (*s*). Then from both components, we construct the feature matrix of user behaviours features and user tweets:

9 $$ {p = p_{1},p_{2}, .... , p_{M}} \in {\mathbb{R}^{1d \times M}} $$

10 $$ s = s_{1},s_{2}, .... , s_{n} \in {\mathbb{R}^{2d \times n}} $$

We further unify these components together, which is denoted as [*p*,*s*]. The output of such a network is typically fed to a sigmoid layer for classification:

11 $$ \hat{y} = sigmoid (b_{f}+[{p},{s}]W_{f}) $$

where where $\hat {y}$ is the predicted probability vector with $\hat {y_{0}}$ and $\hat {y_{1}}$ indicate the predicted probability of label being 0 (not depressed) and 1 (depressed user) respectively. Then, we aim to minimize the cross-entropy error for each user with ground-truth label *y*:

$$ \text{Loss} = - \sum\limits_{i} y_{i} \cdot \log {\hat{y}}_{i} $$

where $\hat {y}_{i}$ is the predicted probability and *y*_*i*_ is the ground truth label (either depression or non-depression) user.

### Explainability

We aim to select user tweets that can explain why a user is depressed. As they provide a reasonable explanation, they should also help detect depression. The hierarchical attention that we explained in the previous sections in the word and tweet encoding is a suitable mechanism for giving high weights of user tweets representations. Besides, the explainability degree of user tweets are learned through the attention weight. Since varied words have different weights in each tweet based on the attention map, it indicates that our model can extract important and long-range contextual information from a tweet. Generally, the attention map of our model can select the most contributed words that identify a depressed and their corresponding tweets. Therefore, user tweets with high attention weight are essential and likely explain why a user is depressed.

## Experiments and results

In this section, we present the experimental evaluation to validate the performance of **MDHAN**. First will we will introduce datasets and evaluation Metrics and experimental settings, followed by the experimental results.

### Comparative methods

We compare our model with the following classification methods:

- **MDL: Multimodal Dictionary Learning Model** is to detect depressed users on Twitter [44]. They use dictionary learning to extract latent data features and sparse representations of a user.
- **SVM: Support Vector Machines** is a popular and strong classifier that has been applied on a wide range of classification tasks [21] and it remains a strong baseline.
- **NB: Naive Bayes** is a family of probabilistic algorithms based on applying Bayes’ theorem with the “naive” assumption of conditional independence between instances [33].
- **BiGRU**:We applied **Bidirectional Gated Recurrent Unit** [7] with attention mechanism to obtain user tweets representations, which we then used for user tweets classification.
- **MBiGRU**: Hybrid model based on MLP and BiGRU for multi-aspect features for the user behaviour and the user’s online timeline (posts).
- **CNN**: We utilized **Convolutional Neural Networks** [22] with an attention mechanism to model user tweets, which can capture the semantics of different convolutional window sizes for depression detection.
- **MCNN**: Hybrid model based on MLP and CNN for multi-aspect features for the user behaviour and the user’s online timeline (posts).
- **HAN**: A hierarchical attention neural network framework [63], it used on user posts for depression detection. The network encodes first user posts with word-level attention on each tweet and tweet-level attention on each user post.
- **MDHAN**: The proposed model in this paper.

### Datasets

Recent research conducted by Shen et al. [44] is one such work that has collected large-scale data with reliable ground truth data, which we aim to reuse. To exemplify the dataset further, the authors collected three complementary data sets, which are:

- Depression data set: Each user is labelled as depressed, based on their tweet content between 2009 and 2016.
- Non-depression data set: Each user is labelled as non-depressed and the tweets were collected in December 2016.
- Depression-candidate data set: The authors collected are labelled as depression-candidate, where the tweet was collected if contained the word “depress”.

Data collection mechanisms are often loosely controlled, impossible data combinations, for instance, users labelled as depressed but have provided no posts, missing values, among others. After data has been crawled, it is still not ready to be used directly by the machine learning model due to various noise still present in data, which is called the “raw data”. The problem is even more exacerbated when data has been downloaded from online social media such as Twitter because tweets may contain spelling and grammar mistakes, smileys, and other undesirable characters. Therefore, a pre-processing strategy is needed to ensure satisfactory data quality for computational modal to achieve reliable predictive analysis.

To further clean the data we used Natural Language processing ToolKit (NLTK). This package has been widely used for text pre-processing [19] and various other works. It has also been widely used for removing common words such as stop words from text [11, 41]. We have removed the common words from users tweets (such as “the”, “an”, etc.) as these are not discriminative or useful enough for our model. These common words sometimes also increase the dimensionality of the problem which could sometimes lead to the “curse-of-dimensionality” problem and may have an impact on the overall model efficiency. To further improve the text quality, we have also removed non-ASCII characters which have also been widely used in literature [64].

Pre-processing and removal of noisy content from the data helped get rid of plenty of noisy content from the dataset. We then obtained high-quality reliable data which we could use in this study. Besides, this distillation helped reduce the computational complexity of the model because we are only dealing with informative data which eventually would be used in modelling. We present the statistics of this distilled data below:

- Number of users labelled positive tweets: 5899.
- Number of tweets from positive users: 508786.
- Number of users labelled negative: 5160.
- Number of tweets from negative users: 2299106.

To further mitigate the issue of sparsity in data, we excluded those users who have posted less than ten posts and users who have less than 5000 followers, therefore we ended up with 2159 positive users and 2049 negative users.

For our experiments, we have used the datasets as mentioned in section (3). They provide a large scale of data, especially for labelled negative and candidate positive, and in our experiments, we used the labelled dataset. We preprocess the dataset by excluding users who have their posting history comprising of less than ten posts or users with followers more than 5000, or users who tweeted in other than English so that we have sufficient statistical information associated with every user. We have thus considered 4208 users (51.30% depressed and 48.69% non-depressed users) as shown in Table 1. For evaluation purposes, we split the dataset randomly into training (80%) and test (20%), and we have reported our experimental results after performing five fold cross-validation.

**Table 1:** Summary of labelled data used to train MDHAN model

Description	Depressed	Non-depressed
Numer of users	2159	2049
Numer of tweets	447856	1349447

### Experimental setting and evaluation metrics

For parameter configurations, the word embeddings are initialized with the Glove [38] with a dimension of 100 on the training set of each dataset to initialize the word embeddings of all the models, including baselines. The hidden dimension has been set to 100 in our model and other neural models, also, the dropout is set to 0.5. All the models are trained to use use the Adam optimization algorithm [24] with a batch size of 16 and an initial learning rate of 0.001. Finally, we trained our model for 10 iterations, with a batch size of 16. The number of iterations was sufficient to converge the model and our experimental results further cement this claim where we outperform existing strong baseline methods, and the training epoch is set to 10. We used python 3.6.3 and Tensorflow 2.1.0 to develop our implementation. We rendered the embedding layer to be not trainable so that we keep the features representations, e.g., word vectors and topic vectors in their original form. We used one hidden layer and a max-pooling layer of size 4 which gave a better performance in our setting.

Finally, we employ traditional popular metrics such as precision, recall, F1, and accuracy based on the confusion matrix to evaluate our model. A confusion matrix is a sensational matrix used for evaluating classification performance, which is also called an error matrix because it shows the number of wrong predictions versus the number of right predictions in a tabulated manner.

### Experimental results

In our experiments, we study our model attributes including the quantitative performance of our hybrid model. For the multi-aspect features and user’s timeline semantic features attribute, we will use both these attributes jointly. After grouped user behaviour in social media into a multi-aspect attribute, we evaluate the performance of the model. First, we examine the effectiveness of using the multi-aspect features only for depression detection with different classifiers. Second, we showed how the model performance increased when we utilize multi-aspect features with hierarchical attention network MDHAN. We summarise the results in Table 2 as follows:

- Naive Bayes obtain the lowest F1 score, which demonstrates that this model has less capability to classify tweets when compared with other existing models to detect depression. The reason for its poor performance could be that the model is not robust enough to sparse and noisy data.
- MDL model outperforms SVM, NB and BiGRU, and obtains better accuracy than these three methods. Since this is a recent model specially designed to discover depressed users, it has captured the intricacies of the dataset well and learned its parameters faithfully leading to better results.
- we can observe the evolving when we integrate The multi-aspect features with user posts and that better helped to analyze a user that seems to be depressed as shown in the performance of MBiGRU, MCNN MDHAN.
- We can see our proposed model MDHAN improved the depression detection up to 10% on F1-Score, compared to MDL model and 5% compared to HAN model. This suggests that our model outperforms a strong model. The reason why our model performs well is primarily that it leverages a rich set of features which is jointly learned in the estimation of the consolidated parameters resulting in a robust model.
- Furthermore, MDHAN achieved the best performance with 89% in F1, indicating that combining HAN with multi-aspect strategy for user timeline semantic features strategy is sufficient to detect depression in Twitter. We can also deduce from the table that our model consistently outperforms all existing and strong baselines.

**Table 2:** Performance comparison of MDHAN against the baselines for depression detection on [44] dataset

Matric	SVM	NB	MDL	BiGRU	MBiGRU	CNN	MCNN	HAN	MDHAN
Accuracy	0.644	0.636	0.787	0.764	0.786	0.806	0.871	0.844	0.895
Precision	0.724	0.724	0.790	0.766	0.789	0.817	0.874	0.870	0.902
Recall	0.632	0.623	0.786	0.762	0.787	0.804	0.870	0.840	0.892
F1-score	0.602	0.588	0.786	0.763	0.786	0.803	0.870	0.839	0.893

### Comparison and discussion

**Fig. 4:**
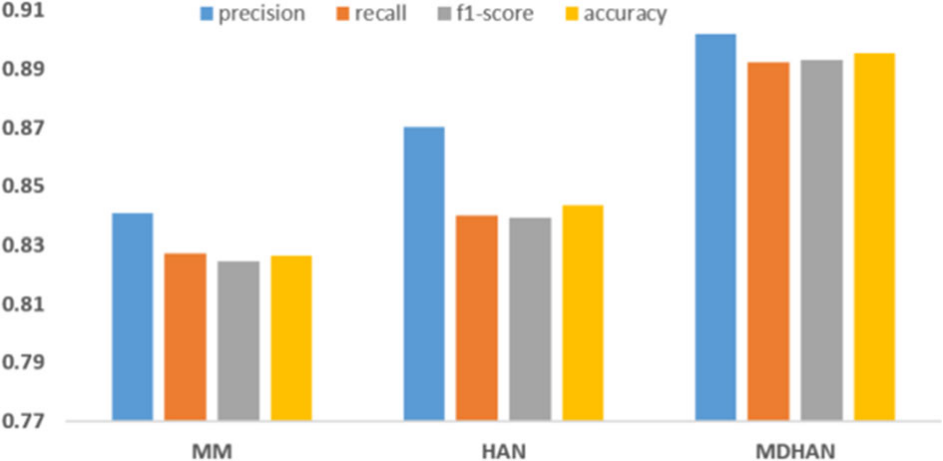
Effectiveness comparison between MDHAN with different attributes

**Fig. 5:**
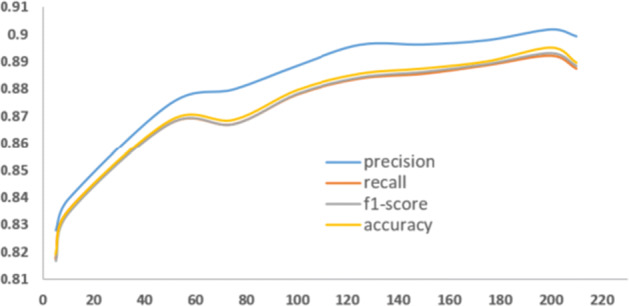
Model vs number of tweets

To get a better look at our model performance, We have compared the effectiveness of each of the two attributes of our model. Therefore, we test the performance of the model with a different attribute, we build the model to feed it with each attribute separately and compare how the model performs. First, we test the model using only the multi-aspect attribute, we can observe in Figure 4 the model perform less optimally when we used **M** LP for **M** ulti-aspect features (MM). In contrast, the model performs better when we use only HAN with word embedding attributes. This signifies that extracting semantic information features from user tweets is crucial for depression detection. Thus, we can see the MDHAN model performance increased when combined both MM and HAN, and outperforms when using each attribute independently. One of the key parameters in MDHAN is the number of tweets for each user; we eventually observed that MDHAN reached optimal performance when using 200 tweets as the maximum number of tweets. Figure 5 illustrates the performance of our model concerning the number of tweets.

**Fig. 6:**
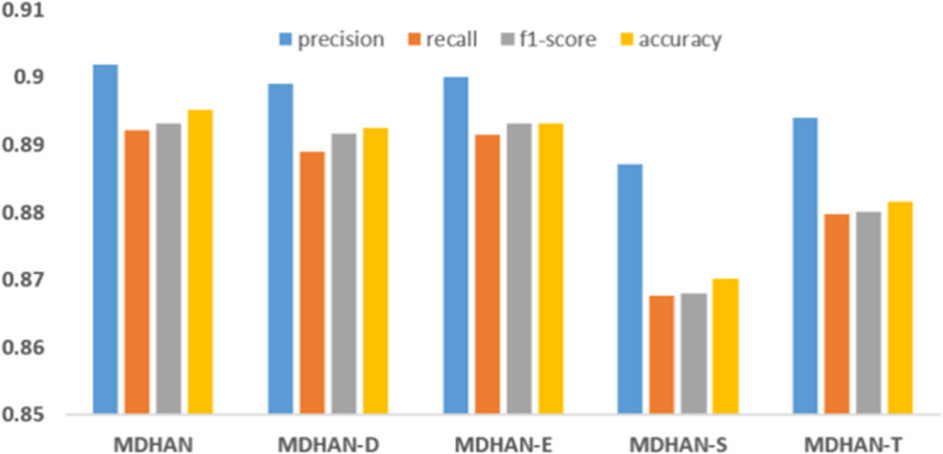
Comparisons of various attributes

**Fig. 7:**
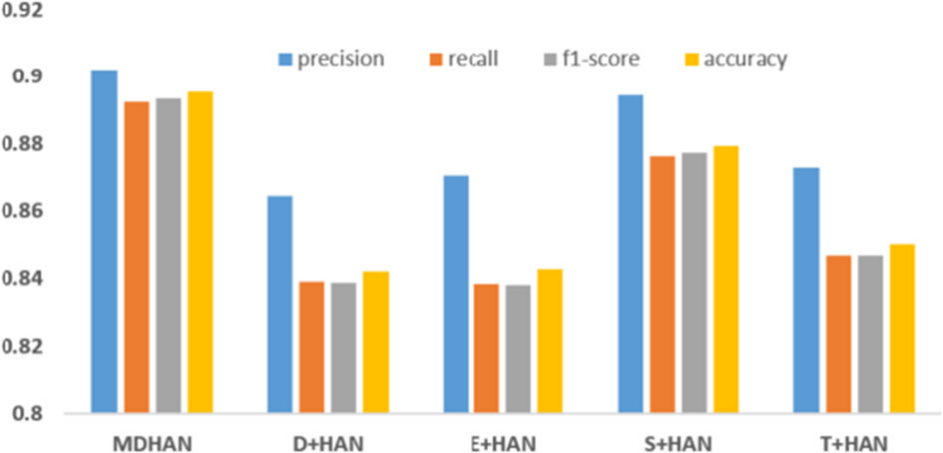
Comparison of various use of attributes

To further analyze the role played by each aspect features and contribution of the user behavioural attributes and user posts attribute, we removed the four aspects separately as following: the domain-specific feature and denote as *MDHAN - D*, emotion feature and denote as *MDHAN - E*, the social network feature and denote this model as *MDHAN - S* and topic feature which we denote as *MDHAN - T*. We can see in Figure 6 that our model performance deteriorates as we remove the topic feature from the MDHAN model and degrades more without the social network features. To dive deeper and understand the effectiveness of each aspect, we combine each aspect separately with HAN and denote them respectively as following: *D+HAN*, *E+HAN*, *S+HAN* and *T+HAN*. As shown in Figure 7, we could see that MDHAN with four aspects outperforms the others, which means that each aspect does contribute to depression detection.

### Case study

**Fig. 8:**
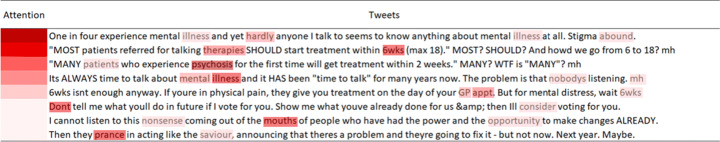
Explainability via visualization of attention score in MDHAN

To illustrate the importance of MDHAN for explaining depression detection results, we visualize the attention map for an example of a depressed user to show the words and tweets captured by MDAHN in Figure 8. The words and tweet weights are indicated by the red in this example, and the words and tweet are more important by attention weight if the colour is darker. Varied words have different weights in each tweet based on the attention map. It indicates that our model can extract important and long-range contextual information from a tweet. Generally, the attention map of our model can select the most contributed words that identify a depressed user, like mental, patients, therapies and illness, and their corresponding tweets. Tweets containing some words that have not contributed to classifying a depressed user and low attention weight will be neglected, for example, in the figure, we will notice that the first tweet has got the most attention, and the same goes for the words: mental and illness that had the highest weights when determining the prediction of class depression. The figure demonstrates that the attention map gives higher weights to explainable depression tweets; for instance, the tweet *“One in four experience mental illness ... ”* gained the highest attention score among all the user tweets. Moreover, MDAHN can give higher weights to explainable tweets than those interfering and unrelated tweets, which can help select more related tweets and to be a more important feature to detect the depressed user.

**Fig. 9:**
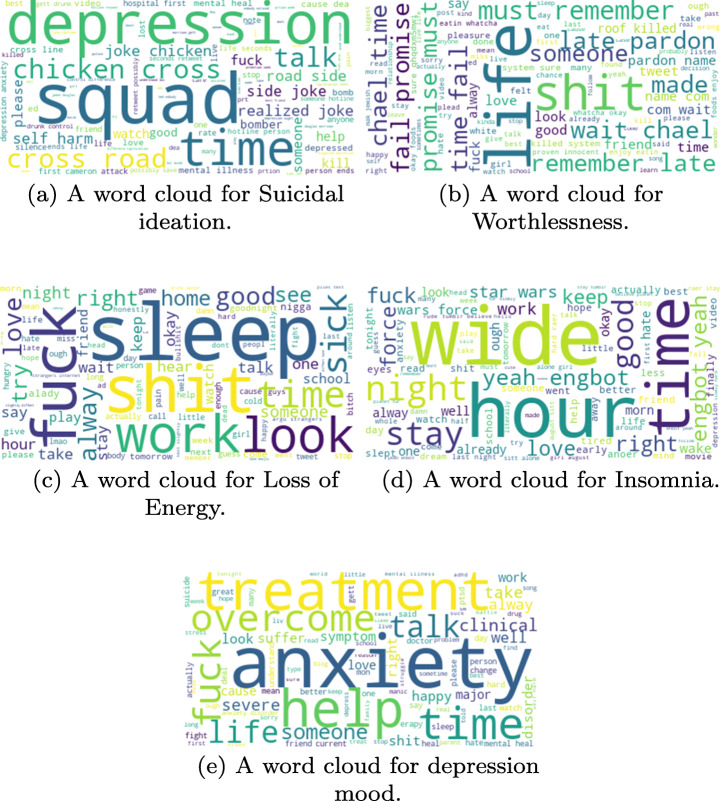
A word cloud depicting the most influencing symptoms

To further investigate the five most influencing symptoms among depressed users, we collected all the tweets associated with these symptoms. Then we created a tag cloud [53] for each of these five symptoms, to determine what are the frequent words and importance that related to each symptom as shown in Figure 9 where larger font words are relatively more important than rest in the same cloud representation. This cloud gives us an overview of all the words that occur most frequently within each of these five symptoms.

## Conclusion

We have proposed explainable Multi-Aspect Depression Detection with Hierarchical Attention Network (MDHAN) for detecting depressed users through social media analysis by extracting features from the user behaviour and the user’s online timeline (posts). We have used a real-world data set for depressed and non-depressed users. Our main contribution is a novel hybrid computational model that can not only effectively model the real-world data but can also help derive explanations from them. We assign the multi-aspect attribute which represents the user behaviour into the MLP and user timeline posts into HAN to calculate each tweet and words’ importance and capture semantic sequence features from the user timelines (posts). Our model shows that training this hybrid network improves classification performance and identifies depressed users outperforming other strong methods and ensures adequate evidence to explain the prediction. In the future, We will analyze users’ tweets by considering topics and sentiments simultaneously to provide supporting evidence for each Depression DSM-IV criteria. Moreover, we will go beyond social media content and use URLs, images, and a mix of short and long user-generated content with traditional web pages. This would help give more contextual knowledge to the model that will help us focus on a task where our model not only detects depression but also automatically suggests the possible diagnosis.

## Appendix: A

List of depression symptoms as per DSM-IV:

1. Depressed mood.
2. iminished interest.
3. Weight or appetite change
4. Insomnia, hypersomnia.
5. Psychomotor retardation, psychomotor impairment.
6. Fatigue or loss of energy
7. Feelings worthlessness, guilt.
8. Diminished ability to think, indecisiveness.
9. Suicidal tendency.

## Appendix: B

List of antidepressant medicine names

**Table:**

Citalopram	Celexa	Cipramil	Escitalopram	Lexapro	Cipralex
Fluoxetine	Prozac	Sarafem	Fluvoxamine	Luvox	Faverin
Paroxetine	Paxil	Seroxat	Sertraline	Zoloft	Lustral
Desvenlafaxine	Pristiq	Duloxetine	Cymbalta	Levomilnac.	Fetzima
Milnacipran	Ixel	Savella	Venlafaxine	Effexor	Vilazodone
Viibryd	Vortioxetine	Trintellix	Nefazodone	Dutonin	Nefadar
Serzone	Trazodone	Desyrel	Atomoxetine	Strattera	Reboxetine
Edronax	Teniloxazine	Lucelan	Metatone	Viloxazine	Vivalan
Bupropion	Wellbutrin	Amitriptyline	Elavil	Endep	Trifluoperazine
Amioxid	Ambivalon	Equilibrin	Clomipramine	Anafranil	Desipramine
Norpramin	Pertofrane	Dibenzepin	Noveril	Victoril	Dimetacrine
Istonil	Dosulepin	Prothiaden	Doxepin	Adapin	Sinequan
Imipramine	Tofranil	Lofepramine	Lomont	Gamanil	Melitracen
Dixeran	Melixeran	Trausabun	Nitroxazepine	Sintamil	Nortriptyline
Pamelor	Aventyl	Noxiptiline	Agedal	Elronon	Nogedal
Opipramol	Insidon	Pipofezine	Azafen	Azaphen	Protriptyline
Vivactil	Trimipramine	Surmontil	Amoxapine	Asendin	Maprotiline
Ludiomil	Mianserin	Tolvon	Mirtazapine	Remeron	Setiptiline
Tecipul	Mianserin	mirtazapine	setiptiline	Isocarboxazid	Marplan
Phenelzine	Nardil	Tranylcyp.	Parnate	Selegiline	Eldepryl
Zelapar	Emsam	Caroxazone	Surodil	Timostenil	Metralindole
Inkazan	Moclobemide	Aurorix	Manerix	Pirlindole	Pirazidol
Toloxatone	Humoryl	Eprobemide	Befol	Minaprine	Brantur
Cantor	Bifemelane	Alnert	Celeport	Agomelatine	Valdoxan
Esketamine	Spravato	Ketamine	Ketalar	Tandospirone	Sediel
Tianeptine	Stablon	Coaxil	Indeloxazine	Elen	Noin
Medifoxamine	Clédial	Gerdaxyl	Oxaflozane	Conflictan	Pivagabine
Tonerg	Ademetionine	Aurorix	SAMe	Heptral	Transmetil
Samyl	Hypericum per.	St. John’s Wort	SJW	Jarsin	Kira
Movina	Oxitriptan	Kira	5-HTP	Cincofarm	Levothym
Triptum	Rubidium chl.	Rubinorm	Tryptophan	Tryptan	Optimax
Aminomine	Magnesium	Noveril	Solian	Aripiprazole	Abilify
Brexpiprazole	Rexulti	Lurasidone	Latuda	Olanzapine	Zyprexa
Quetiapine	Seroquel	Risperidone	Risperdal	Buspirone	Buspar
Lithium	Eskalith	Lithobid	Modafinil	Thyroxine	Triiodoth.
Minocycline	Amitriptyline	chlordiaz.	Limbitrol	Parmodalin	Aurorix
Perphenazine	Etafron	Flupentixol	Melitracen	Deanxit	Surodil
